# Supplementary material for: In-Depth Characterization of Layer 5 Output Neurons of the Primary Somatosensory Cortex Innervating the Mouse Dorsal Spinal Cord
Source: Cereb Cortex Commun. 2020 Aug 20;1(1):tgaa052. doi: 10.1093/texcom/tgaa052 (PMC8152836; doi:10.1093/texcom/tgaa052)
Supplement: Frezel_et_al_Cerebral_Cortex_10082020_SUPPLEMENTARY_tgaa052 [file frezel_et_al_cerebral_cortex_10082020_supplementary_tgaa052.docx]

**Supplementary figures**

**
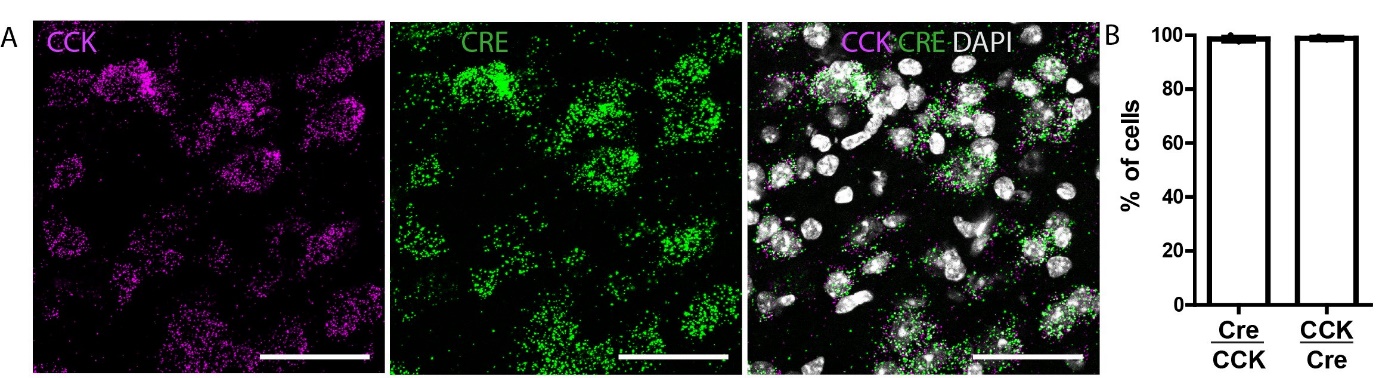
**

**Fig. S1 Overlap between CCK and Cre mRNA expression in CCK^Cre^ mice:** (A) Double ISH showing the colocalization of Cre with CCK mRNA. (B) Quantification of (A): 98.6 ± 1.2% CCK positive neurons are Cre positive and 98.8 ± 0.7% Cre positive neurons are CCK positive (n = 3 mice; 1087 neurons). Error bars represent ± SEM. Scale bars: 50 µm.

**
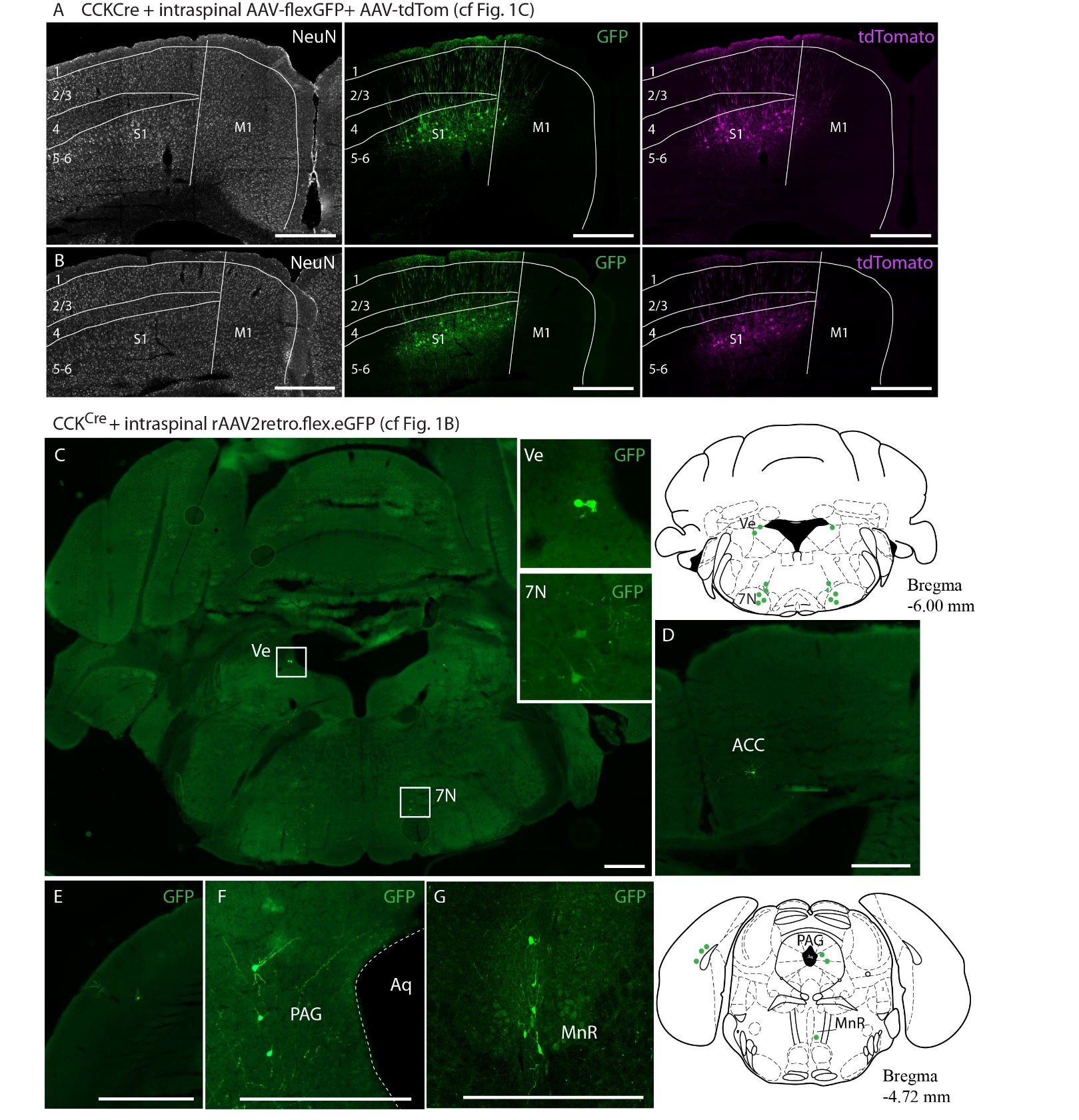
**

**Fig. S2: Labelling S1-CST and other brain neurons in CCK^Cre^ mice.** (A-B) Labelling of S1-CST neurons by Cre-dependent eGFP and Cre-independent tdTomato (n = 4, cf. Fig. 1C) fluorescent proteins encoded by rAAV2retro vectors. Localization of the labelled neurons compared to NeuN immunostaining. Cortical Layer and the demarcation between S1 and M are indicated. (C-G) Brain areas labelled with eGFP positive neurons after intraspinal injection of rAAV2retro.flex.eGFP in CCK^Cre^ mice (related to Fig. 1B). 7N: facial nuclei, ACC: anterior cingulate cortex, MnR: median raphe nucleus, PAG: Periaqueductal grey, Aq: aqueduct, S1hl: somatosensory cortex, hindlimb area. Scale bars: 500 µm.


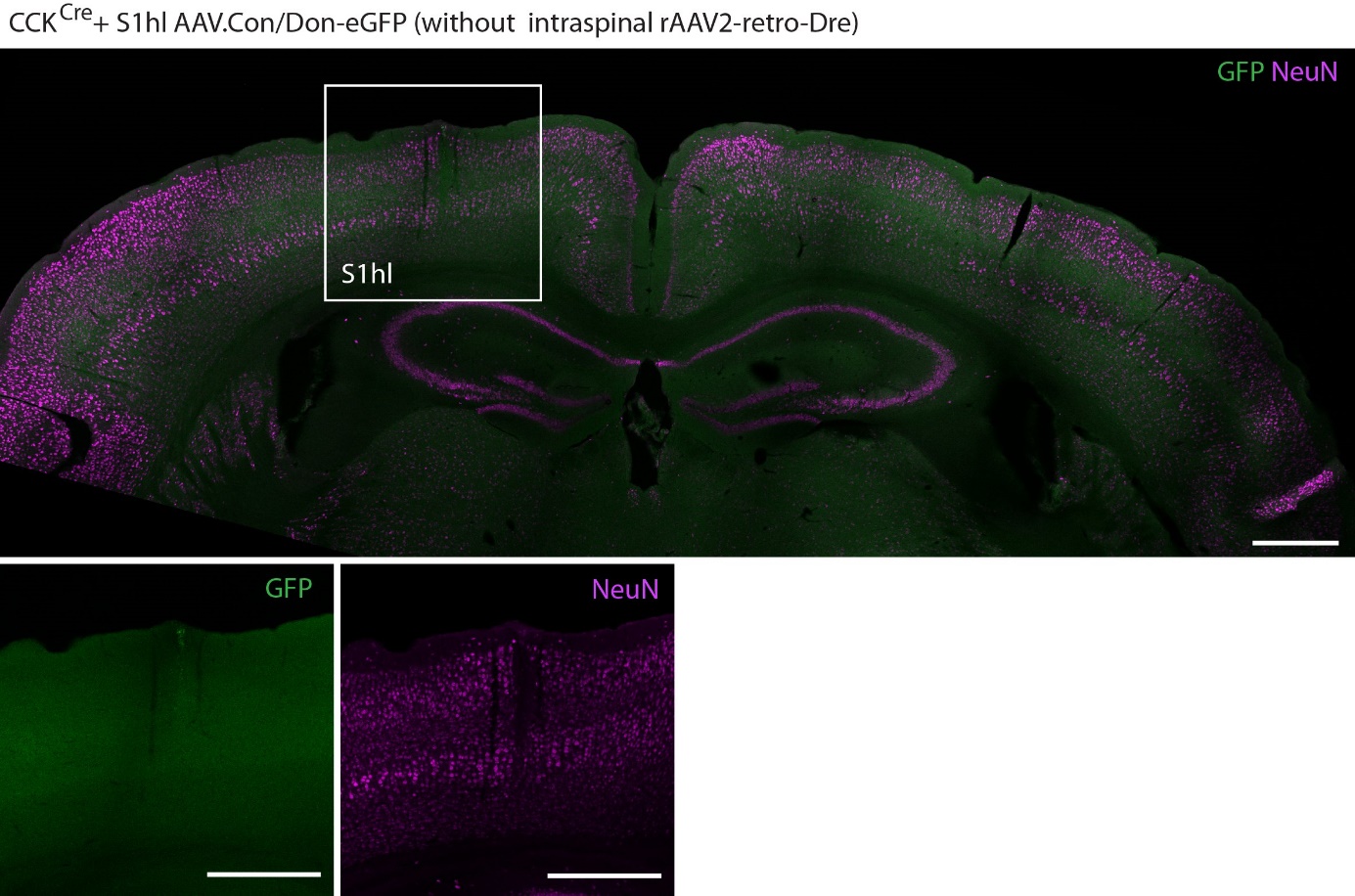


**Fig. S3:** **Expression control of Cre and Dre-dependant rAAVs (related to Fig. 2B).** No GFP labelled neurons in S1 after injection of AAV.C_on_/D_on_-eGFP in CCK^Cre^ mice without prior intraspinal injection of rAAV2-retro-Dre. S1hl: somatosensory cortex, hindlimb area. Scale bars: 500 µm.

**
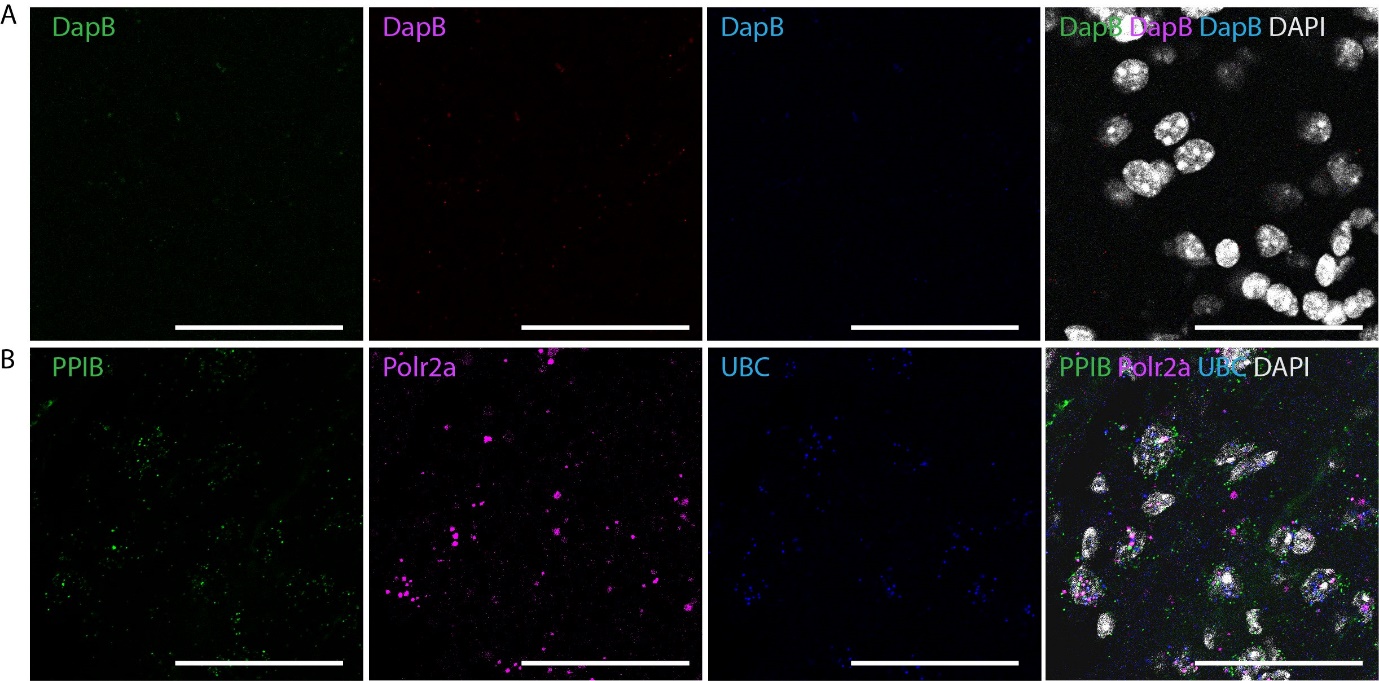
**

**Fig. S4 (related to Fig. 3): Controls for the multiplex ISH in GFP-labelled CCK^Cre^ neurons.** (A) Triple ISH negative control showing signals for the marker *DapB*. (B) Triple ISH positive control showing signals for the markers *PPIB*, *Polr2a* and *UBC*. (n = 3 mice). Scale bars: 50 µm.

**
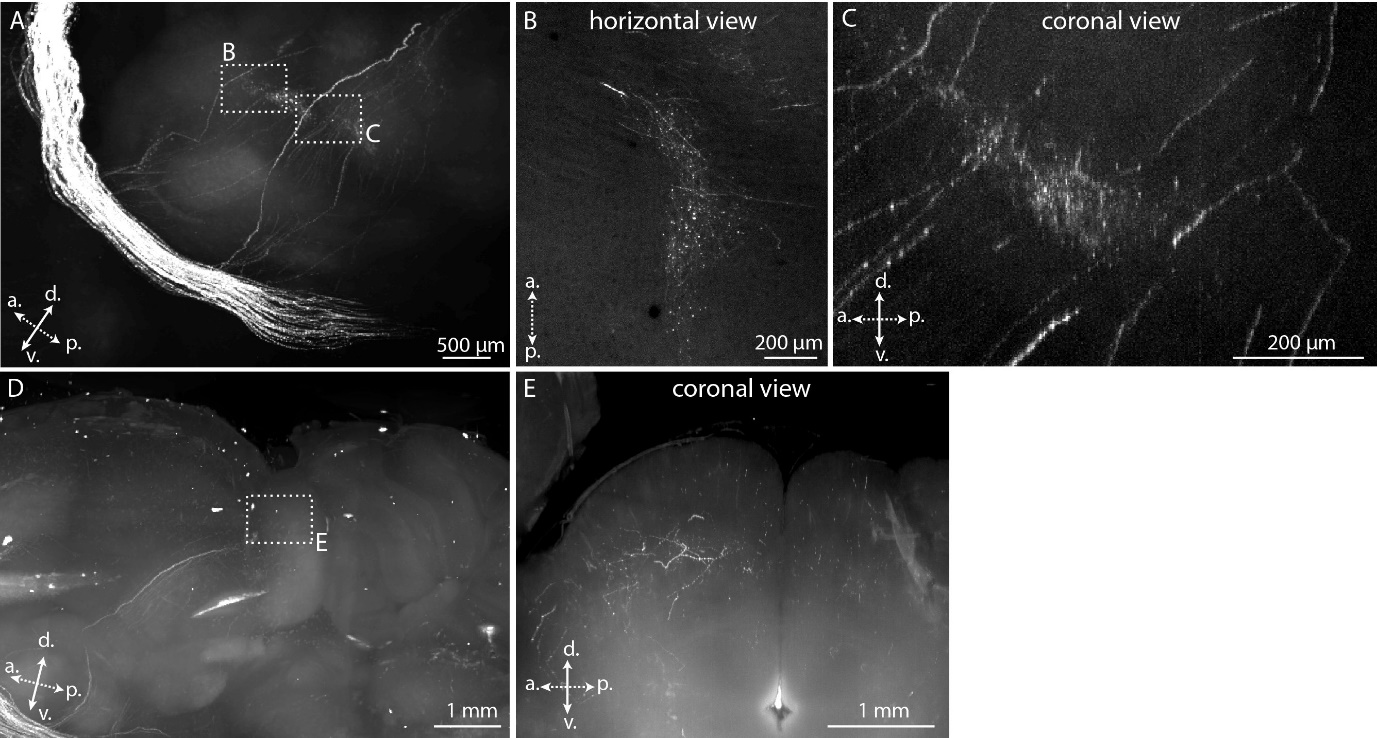
**

**Fig. S5 (related to Fig. 4):** **Detail of the CST collaterals in the thalamus.** (A) Collaterals branching of from the main CST towards the thalamus (Fig. 4C), showing 2 termination areas (B) and (C). (C) Collaterals terminating in tectal areas. (E) Coronal view of inset from (D). (n = 3 mice). a.: anterior, p.: posterior, d.: dorsal, v.: ventral.


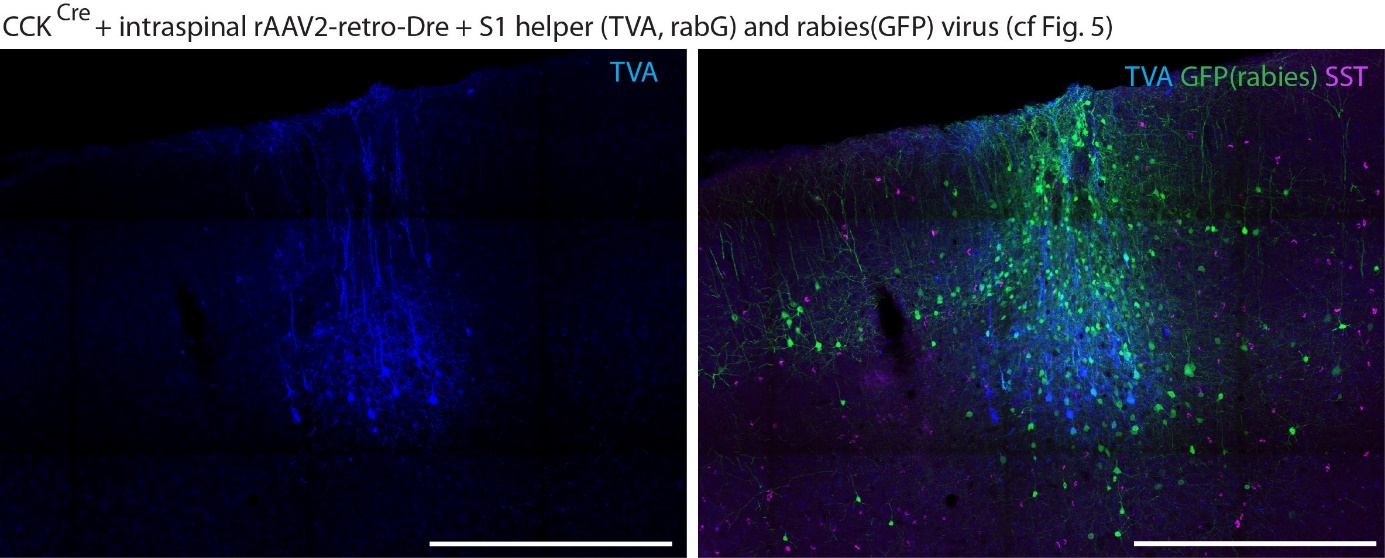


**Fig. S6: TVA labelling of starter cells in retrograde monosynaptic tracing of S1-CST neurons with rabies (related to Fig. 5).** rAAV2retro.Dre was injected into the spinal cord of CCK^Cre^ mice, followed by a Cre-and-Dre-dependent helper virus (TVA, RabG) into S1. Two weeks later the pseudotyped rabies virus was injected into S1. (A) Overview of the labelled neurons in the brain: S1-CST neurons (starter cells) as well as layer 2/3 pyramidal neurons, thalamic sensory relay neurons and layer 5 inhibitory interneurons. Starter cells (S1-CST neurons) are labelled with TVA and GFP. Scale bars: 500 µm.


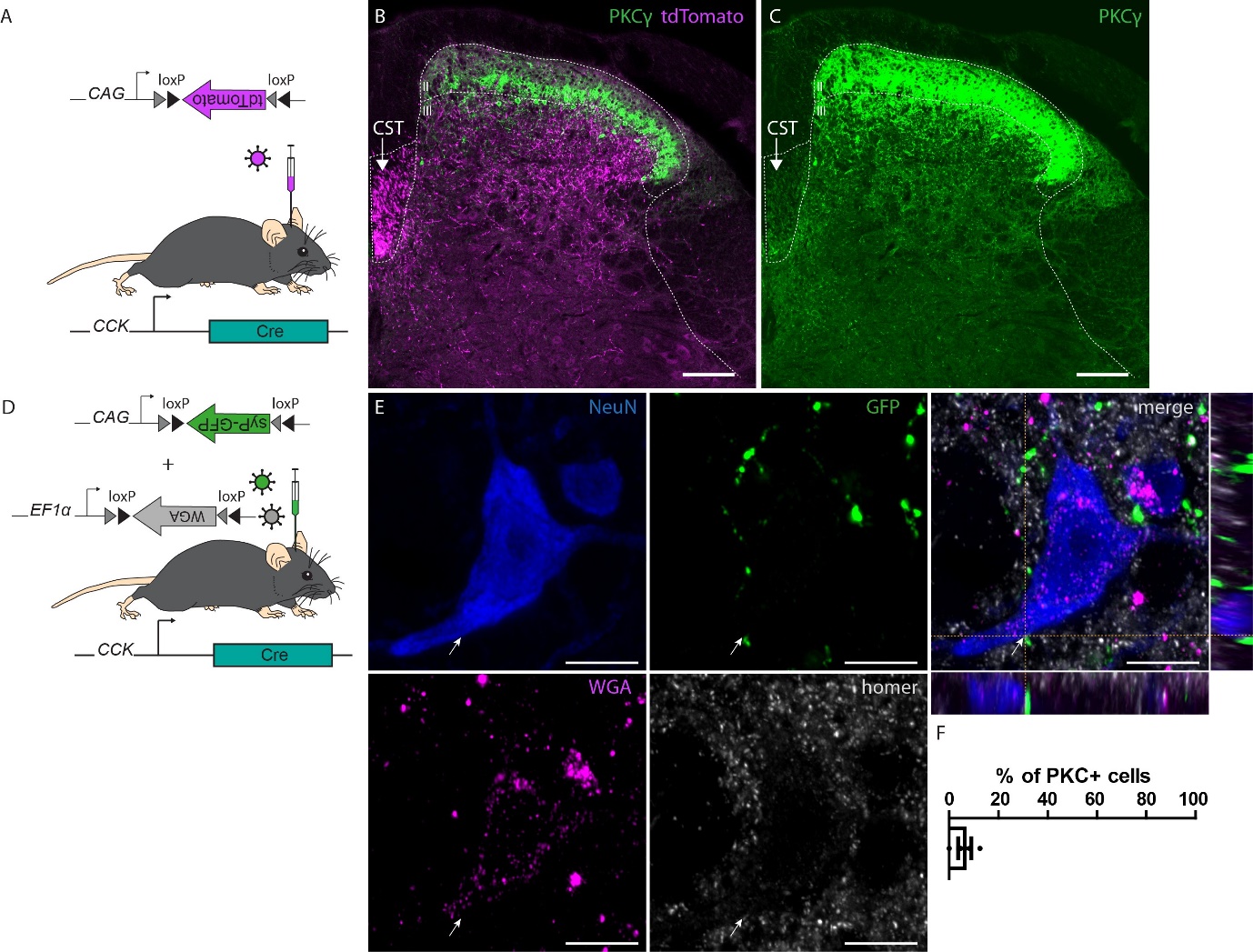


**Fig. S7 (related to Fig. 6)**: **Localisation of S1-CST neurons terminals in the spinal cord.** (A) rAAVs carrying a Cre-dependent tdTomato were injected in the S1hl of CCK^Cre^ mice. (B) Labelling of the CST in the dorsal funiculus of the spinal cord (same sample as in Fig. 6A), contralateral to the brain injection site. CST terminals are preferentially located below the laminae II-III border marked by PKCγ immunoreactivity. (C) The CST is also labelled by PKCγ immunoreactivity. (D) rAAVs carrying a Cre-dependent WGA and a Cre-dependant synaptophysin-eGFP fusion protein transgenes were injected in S1hl of CCK^Cre^ mice. (E) Representative example of a WGA^+^ neuron in close proximity to a GFP^+^ presynaptic terminals of S1-CST neurons after co-injection of rAAVs carrying a Cre-dependent WGA and a Cre-dependant synaptophysin-eGFP fusion protein transgenes in S1hl of CCK^Cre^ mice (from a different animal as the example presented in Fig. 6, n =4). (F) Quantification of the number of PKCγ^+^ neurons receiving direct contacts from eGFP^+^ synaptic terminals (n = 4 mice; 98 neurons). CST: corticospinal tract Error bars represent ± SEM. Scale bars: B-C: 100 µm; E: 10 µm.
